# Neuronal apoptosis inhibitory protein (NAIP) localizes to the cytokinetic machinery during cell division

**DOI:** 10.1038/srep39981

**Published:** 2017-01-06

**Authors:** Francisco Abadía-Molina, Virginia Morón-Calvente, Stephen D. Baird, Fahad Shamim, Francisco Martín, Alex MacKenzie

**Affiliations:** 1 Department of Cell Biology, University of Granada, Granada 18071, Spain; 2 Biomedical Research Centre, University of Granada, Granada 18100, Spain; 3 Children’s Hospital of Eastern Ontario Research Institute, University of Ottawa, Ottawa ON K1H 8L1, Canada; 4 Department of Human DNA Variability, GENYO, Centre for Genomics and Oncological Research, Pfizer, University of Granada, Andalusian Regional Government, Granada, 18016 Spain; 5 Department of Pediatrics, University of Ottawa, Ottawa ON K1H 8L1, Canada

## Abstract

The neuronal apoptosis inhibitory protein (NAIP) is a constituent of the inflammasome and a key component of the innate immune system. Here we use immunofluorescence to position NAIP within the cytokinetic apparatus, contiguous to chromosomal passenger complex (CPC), Centralspindlin, PRC1 and KIF4A. During metaphase, NAIP accumulates in the mitotic spindle poles and is shown in spindle microtubules; in anaphase NAIP is detected in the middle of the central spindle. At the end of cytokinesis, NAIP is localized in the outlying region of the stem body, the center of the intercellular bridge formed between daughter cells prior to cellular abscission. We also describe the sustained presence of NAIP mRNA and protein throughout the cell cycle with a significant increase observed in the G2/M phase. Consistent with a role for NAIP in cytokinesis, NAIP overexpression in HeLa cells promotes the acquisition of a multinuclear phenotype. Conversely, NAIP siRNA gene silencing results in an apoptotic lethal phenotype. Our confocal and super resolution stimulated-emission-depletion (STED) examination of mammalian cell cytokinesis demonstrate a potential new role for NAIP in addition to anti-apoptotic and innate immunology functions.

Cytokinesis is the final step in the cell cycle, by which dividing cells physically separate into two cells following mitotic sister chromatid segregation. Soon after anaphase is initiated, the mitotic spindle reorganizes in an array of antiparallel microtubules to form the central spindle at the cell equator; at the same time, the actomyosin contractile ring organizes along the cleavage furrow in the cell cortex beneath the plasma membrane. These two processes, formation of the central spindle and organization of the contractile ring, define the division plane; subsequently, activation of the contractile ring gradually constricts the dividing cell. Ingression of the cleavage furrow progressively compresses the central spindle into a structure first described in 1891 by Walther Flemming, the intercellular bridge. Often referred as the midbody, the intercellular bridge constitutes the last physical link between the daughter cells and serves as the platform for abscission, the final step in cytokinesis by which the two daughter cells effectively complete partition by plasma membrane fission^1^,^2^,^3^.

The neuronal apoptosis inhibitory protein (NAIP) is the founding member of the mammalian inhibitor of apoptosis protein (IAP) family^4^,^5^, comprised of three zinc-binding baculovirus IAP repeat (BIR) domains and, uniquely among IAPs, a nucleotide-binding and oligomerization (NOD) domain and a leucine rich repeat (LRR) domain; NAIP is therefore also a member of the NOD-like receptor (NLR) superfamily^6^,^7^. BIR domains can also mediate an extensive range of protein-protein interactions, initially considered only a suppressor of programmed cell death^5^,^8^,^9^, more recently, NAIP has emerged as an important regulator of innate immune signalling^10^. NLRs are intracellular sensors for pathogen- and damage-associated molecular patterns (PAMPs and DAMPs); as such NAIP is involved with the intracellular recognition of flagellin, the main structural component of the bacterium flagellum, and the bacterial needle and rod proteins^11^,^12^,^13^,^14^, evolutionary conserved components of bacterial type-III secretion systems. NAIP participates in the formation of the NLRC4 inflammasome^15^, a signalling platform that upon PAMP-ligand binding recruits and activates caspase-1, a proteolytic enzyme that processes the proforms of interleukin-1*β* and interleukin-18 cytokines for extracellular secretion.

NAIP, originally cloned as a candidate for the neurodegenerative disorder spinal muscular atrophy (SMA)^4^, has also been investigated in other neurodegenerative disorders such as Alzheimer’s disease, Parkinson’s disease and multiple sclerosis^16^,^17^,^18^,^19^. Additionally, NAIP has been studied in some cancers^20^,^21^,^22^ and recently, has been proposed in a mouse model to protect against colonic tumorigenesis^23^. The region of the human chromosome that encodes NAIP (5q13) has been described as highly variable^24^,^25^ and rich in gene copy number variation. Consistent with its role in innate immunology, a higher copy number of the full NAIP gene has been shown to protect against *Legionella pneumophila* infection in human populations^26^; given its antiapoptostic role, it has also been inversely related with the clinical severity of SMA^27^.

The protein required for cytokinesis 1 (PRC1), kinesin KIF4A, the chromosomal passenger complex (CPC) and Centralspindlin, are all essential structural and functional components of cytokinesis. The microtubule stabilizers PRC1 and KIF4A bind between antiparallel microtubules to either bundle (PRC1)^28^,^29^ or prevent tubulin polymerization at the plus ends of microtubules in the central spindle (KIF4A)^30^ conferring stability to the overlapping array of microtubules at the division plane level. CPC is a hetero-tetramer composed of Aurora B, the inner centromere protein (INCENP), Survivin and Borealin^31^,^32^. CPC coordinates appropriate chromosome segregation during cytokinesis by functioning at different locations at different stages of mitosis. Centralspindlin^33^,^34^, a hetero-tetramer which consists of two dimers of the Rho-family GTPase activating protein (GAP) MgcRacGAP, and the kinesin motor protein KIF23 (also known as MKLP1), is involved in the bundling of central spindle microtubules serving as the link between the central spindle and the plasma membrane during cytokinesis^35^.

In this detailed microscopic analysis we report the unanticipated co-localization of NAIP with the cytokinetic machinery throughout all stages of the final step in cell division in a pattern distinct from previously characterized regulators of cytokinesis. The molecular dissection of the novel role for NAIP in cytokinesis suggested by our data may lead to a better understanding of this critical cellular process.

## Results

### Confocal microscopy

**Figure 1:**
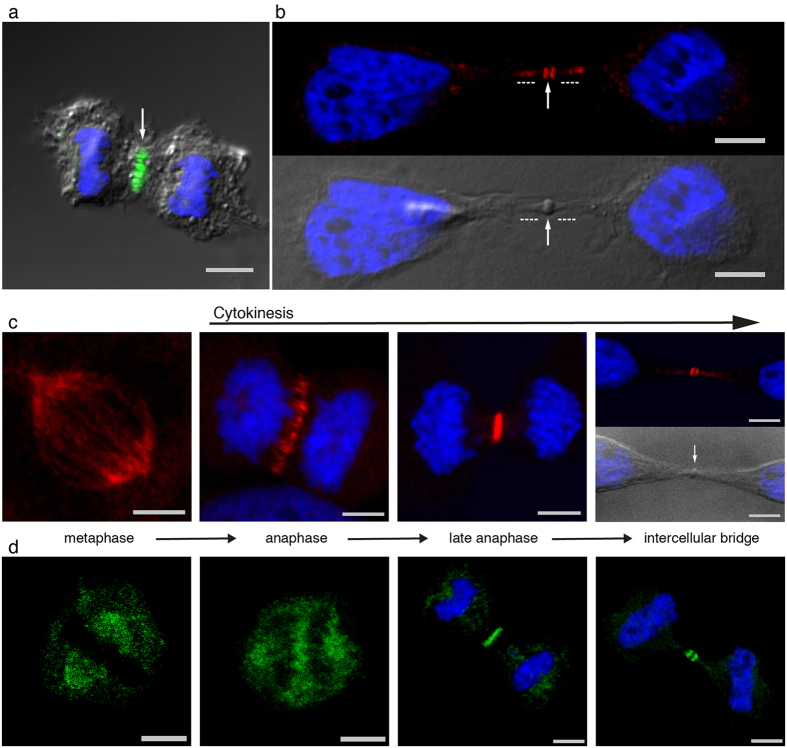
NAIP dynamics in cytokinesis. Confocal differential interference contrast, nuclear Hoechst staining and NAIP immunofluorescence channels merged accordingly. In the metaphase images and in one anaphase image the Hoechst channel has been omitted for clarity. In (**a**),(**b**) and (**c**) NAIP immunostaing was performed with an antibody mapping to 13 C-terminal amino acids of human NAIP (abcam, ab25968). (**a**) Anaphase showing NAIP immunofluorescence in the central spindle (arrow), the staining corresponds with the antiparallel microtubule plus-ends intersection area at the central spindle. (**b**) Intercellular bridge showing NAIP immunofluorescence in the outer regions of the stem body (arrow) and in the flanking zone of the stem body (dashed lines). The intercellular bridge can be subdivided into three known areas; the central stem body, the upper and lower portions of stem body, also referred as the bulge, and the flanking region^40^. (**c**) NAIP dynamics during cytokinesis. NAIP accumulates in the spindle poles in metaphase and is also shown in spindle microtubules, later, once cytokinesis has started, NAIP is restricted to the middle of the central spindle gradually concentrating along the cell division plane as the cleavage furrow progressively constricts the dividing cell. Near cytokinesis completion, NAIP is present in the outflanking regions of the stem body (arrow) in the center of the intercellular bridge. (**d**) NAIP in cytokinesis demonstrated with a different NAIP antibody. NAIP immunofluorescence performed with a commercial polyclonal antibody, epitope mapping to the first 100 residues of human NAIP (abcam, ab98020), showing a pattern as described in (**c**). Bar, 5 *μ*m.

An earlier study of NAIP distribution in human tissues^36^ showed a particularly intense staining in the small intestine epithelium. Based on these results, the further characterization of NAIP by confocal microscopy in the human epithelial carcinoma cell line HeLa, was undertaken and unexpectedly demonstrated the presence of NAIP in the central spindle (Fig. 1a) and in the intercellular bridge (Fig. 1b). A representative view of NAIP dynamics throughout cytokinesis in HeLa cells is shown in Fig. 1c and in Fig. 1d using two commercial human-NAIP antibodies. Equivalent NAIP dynamics are shown employing the main human-NAIP antibody used in this study and a commercially available mouse-NAIP antibody in mouse adipose-derived mesenchymal stem cells, mASCs, (Supplementary Fig. S1). Three additional custom made hybridoma culture supernatants directed at different epitopes which, while not detecting endogenous NAIP, also demonstrated NAIP along cytokinesis in HeLa cells transduced with NAIP-lentiviral particles (Supplementary Fig. S2). Consequently, a series of double immunostainings for NAIP and well defined structural and functional regulators of cytokinesis including the central spindle microtubule stabilizers PRC1 and KIF4A, the mitotic regulator CPC and Centralspindlin was undertaken.

#### NAIP and the central spindle microtubule bundling factors PRC1 and KIF4A

**Figure 2:**
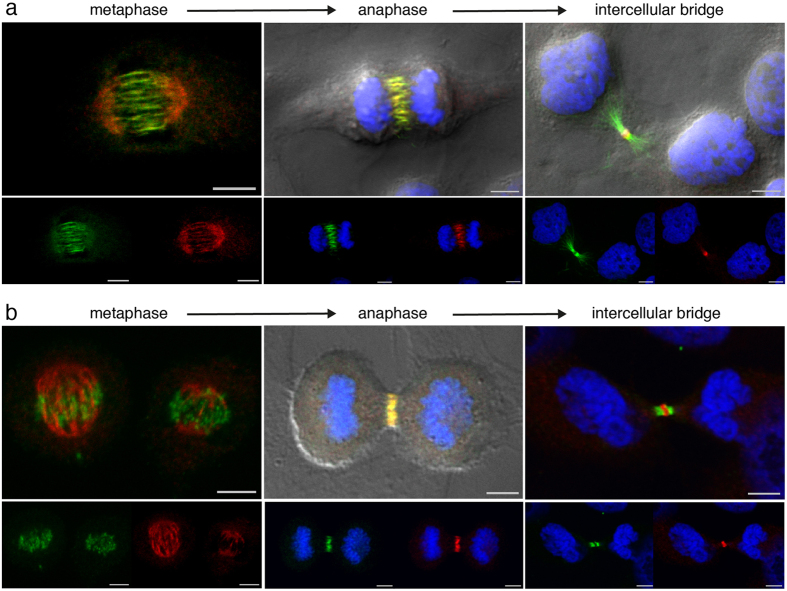
NAIP and the central spindle bundling factors PRC1 and KIF4A. Confocal differential interference contrast, Hoechst DNA staining, PRC1 or KIF4A (Alexa Fluor-488 shown in green) and NAIP abcam ab25968 (Alexa Fluor-568 shown in red), both individual and merged channels. In the metaphase images the Hoechst channel has been omitted for clarity. (**a**) PRC1 and NAIP double immunostaining in metaphase showing a predominant presence of NAIP in the spindle poles and PRC1 clearly decorating spindle microtubules. A complete PRC1 and NAIP central spindle colocalization is shown in anaphase. Once the intercellular bridge is formed, PRC1 and NAIP have segregated into the flanking regions and the stem body respectively. (**b**) KIF4A and NAIP double immunostaining showing a distribution as described in (**a**) but with a minor presence of KIF4A in the two metaphase spindles shown as well as in the intercellular bridge arms. Bar, 5 *μ*m.

As a homodimer, PRC1 binds to the interface between antiparallel microtubules in the central spindle^28^,^29^, the kinesin KIF4A is then targeted to the microtubule overlapping region by PRC1^28^,^30^, with the resulting inhibition of microtubule polymerization, modulating the length of the overlap belt. Other reports suggest that the motor protein KIF4A is required for PRC1 translocation to the plus ends of overlapping microtubules^37^,^38^. Confocal microscopy examination of HeLa cells stained for NAIP and PRC1 or KIF4A (Fig. 2), showed the association of PRC1 and KIF4A with spindle microtubules in metaphase which, with the onset of anaphase, is restricted to the central spindle. NAIP mainly accumulates in the poles of the spindle during metaphase; later in anaphase NAIP colocalizes both with PRC1 and KIF4A in the central spindle microtubules. With the progression of cytokinesis, NAIP is finally restricted to the stem body of the intercellular bridge while PRC1 and KIF4 are present in the arms and flanking zone of the intercellular bridge, respectively.

#### NAIP and the Chromosomal Passenger Complex

**Figure 3:**
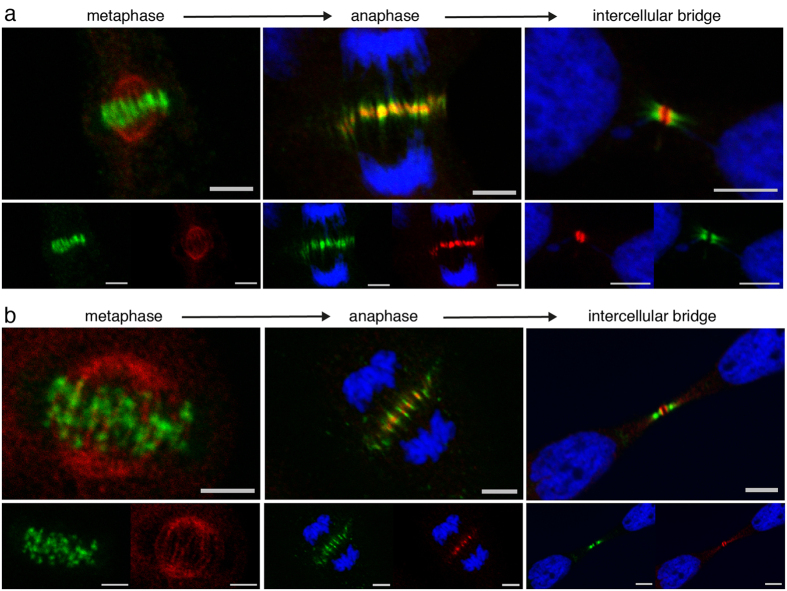
NAIP and the chromosomal passenger complex (CPC). Hoechst DNA staining, Aurora B or INCENP (Alexa Fluor-488 shown in green) and NAIP abcam ab25968 (Alexa Fluor-568 shown in red) individual and merged confocal channels. In the metaphase images the Hoechst channel has been omitted for clarity. (**a**) Aurora B and NAIP double immunostaining: in metaphase Aurora B is localized to the metaphase plate area while NAIP accumulates in the spindle poles, during anaphase Aurora B is localized in the middle of the central spindle while NAIP is present in the centermost zone. In the intercellular bridge, Aurora B is present in the flanking zone and NAIP localizes to the outer area of the stem body. (**b**) INCENP and NAIP double immunostaining showing a pattern as described in (**a**), in metaphase INCENP immunofluorecence clearly depicts mitotic chromosomal centromeres and in anaphase maps to the sides of NAIP immunofluorescence. NAIP and survivin double immunostainings, omitted here for brevity, are shown in the Supplementary information (Fig. S2). Bar, 5 *μ*m.

The extensively studied heterotetrameric CPC corrects improper kinetochore-microtubule attachments, participates in spindle assembly checkpoint and has thus been shown to be central to both cytokinesis progression and mitotic exit^31^,^32^,^39^. We have performed double immunostainings for NAIP and the CPC components: Aurora B, INCENP (Fig. 3) and Survivin (Supplementary Fig. S3). Consistent with previous reports, CPC was initially localized to the centromere of mitotic chromosomes prior to anaphase, thereafter relocating to the central spindle; by the completion of cytokinesis, CPC is shown in the flanking zones of the intercellular bridge^40^. NAIP immunolocalization was as described above with PRC1 and KIF4A, making the transition from poles of the spindle to the central spindle microtubules and finally to the stem body of the intercellular bridge.

#### NAIP and Centralspindlin

**Figure 4:**
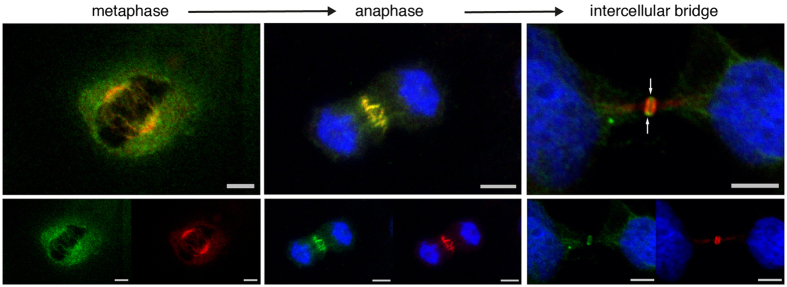
NAIP and Centralspindlin. Hoechst DNA staining, MgcRacGAP (Alexa Fluor-488 shown in green) and NAIP abcam ab25968 (Alexa Fluor-568 shown in red) confocal channels individually and merged. The Hoechst channel has been omitted in the metaphase images for clarity. In metaphase and anaphase, MgcRacGAP and NAIP largely colocalize (orange in the upper merged images), while in the intercellular bridge MgcRagGAP is predominantly shown in the bulge (arrows) and NAIP is detected in the stem body flanks. Bar, 5 *μ*m.

The heterotetrameric Centralspindlin complex is another fundamental component of the cytokinetic apparatus, comprised of a dimer of the Rho-family GAP protein MgcRacGAP and a dimer of the motor protein KIF23. Centralspindlin travels along microtubules towards the central spindle^41^, where it mediates the bundling of antiparallel microtubules during central spindle assembly. The complex also anchors, through the MgcRacGAP C1 domain, the intercellular bridge microtubules to the plasma membrane^35^; a role for the complex in abscission has also been proposed^42^. Double immunostaining for MgcRacGAP and NAIP show colocalization during metaphase in the spindle poles and along spindle microtubules (Fig. 4 metaphase) as well as a very sharp colocalization later in anaphase (Fig. 4 anaphase). While NAIP in the intercellular bridge was typically observed to bracket the stem body, MgcRacGAP defined a distinct circular structure surrounding it (Fig. 4 intercellular bridge) with a stronger presence in the sections more closely apposed to the plasma membrane.

### STED super-resolution microscopy

**Figure 5:**
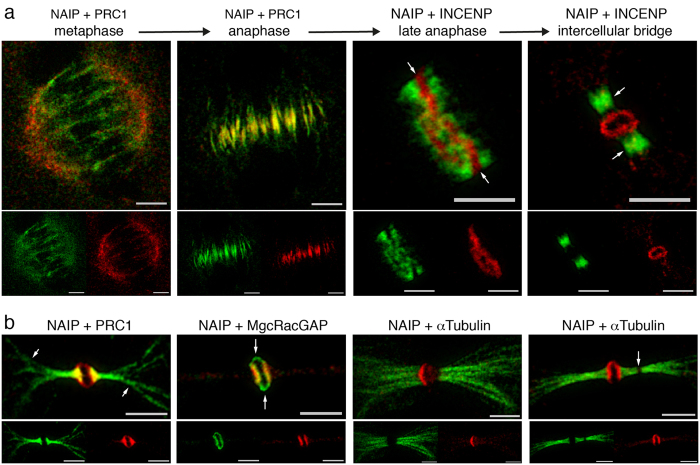
STED microscopy. Dual STED microscopy for NAIP abcam ab25968 (Chromeo-505 fluorescence shown in red) and PRC1, INCENP or *α*-Tubulin (Biotin fluorescence shown in green), STED channels merged accordingly. (**a**) Image series showing the distribution of NAIP at individual phases along the cytokinesis timeline in conjunction with well characterized cytokinesis regulators. Before cytokinesis initiation, in metaphase, NAIP is primarily visualized in the spindle poles, while the microtubule stabilizers (PRC1, KIF4A) and Centralspindlin are observed in spindle microtubules. In anaphase, NAIP immunostaining occupies the center of the central spindle, colocalizes with PRC1, KIF4A, the CPC components and Centralspindlin. Gradual ingression of the cleavage furrow constricts the central spindle into a ring in which NAIP occupies the centermost section (arrows) and colocalizes with MgcRacGAP, while PRC1, KIF4A and CPC have segregated to both sides of NAIP (late anaphase). Then, when the intercellular bridge is completely formed, NAIP is present in the bulge along with MgcRacGAP while the microtubule stabilizers and CPC localize to the intercellular bridge flanking area. (**b**) NAIP + PRC1: NAIP immunofluorescence is shown in the outer stem body area while PRC1 extends on both sides from the intercellular bridge center well beyond the flanking zone and into the nascent daughter cells (arrows). NAIP + MgcRacGAP: Both proteins are shown delimiting the stem body margins, interestingly MgcRacGAP occupies the upper and lower portions of the stem body precisely (arrows). NAIP + *α*-Tubulin: An array of microtubule bundles is shown lengthening at both sides on the intercellular bridge center, an abscission point can be identified as a disruption lacking any immunostaining (arrow). Bar, 2, 5 *μ*m.

Super-resolution microscopy overcomes the resolution limit imposed by the diffraction of light in optical instruments^43^. STED microscopy was therefore used to gain further insight beyond our confocal microscopy results. The results of the dual channel STED microscopy study performed for NAIP and cytokinesis regulators were completely consistent with our confocal microscopy observations (Fig. 5). In metaphase, PRC1 decorates spindle microtubules while most of the NAIP immunofluorescence concentrates in the spindle poles (Fig. 5a-metaphase). In anaphase, with the initiation of cytokinesis, PRC1 staining can be seen exclusively in the central spindle but not the rest of the mitotic spindle; similarly NAIP is shown uniquely in the centermost section of the central spindle but, again, is absent from the rest of the mitotic spindle (Fig. 5a-anaphase). During cleavage furrow ingression and the compaction of the central spindle into a ring, NAIP is precisely observed in the ring center with CPC immediately contiguous to it (Fig. 5a-late anaphase). PRC1, KIF4A, and the CPC components are present in the flanking areas of the intercellular bridge, whereas NAIP is shown in the stem body (Fig. 5a-intercellular bridge). At this stage, a branch of PRC1 immunofluorescence is observed extending well beyond the intercellular bridge and into the nascent daughter cells (Fig. 5b-NAIP + PRC1, Fig. 2a-intercellular bridge). STED microscopy shows MgcRacGAP staining demarcating a round structure in the margins of the stem body, while NAIP is generally observed in a sandwich like configuration, the sides in contact with the flanking region of the intercellular bridge (Fig. 5b-NAIP + MgcRacGAP). Dual STED microscopy analysis for NAIP and *α*-Tubulin (Fig. 5b) clearly showed an extensive array of bundled *α*-Tubulin stained microtubules stretching outwards from the intercellular bridge stem body and, near the completion of cytokinesis, the abscission point as a region lacking any *α*-Tubulin or any of the other cytokinesis regulators under study.

### NAIP expression is sustained along the cell cycle

**Figure 6:**
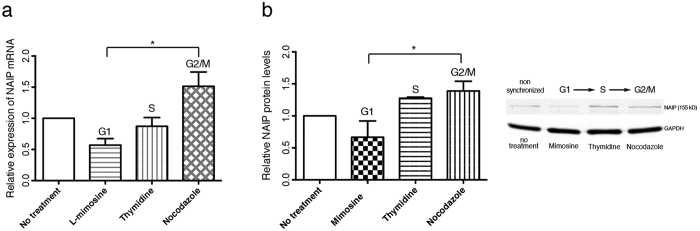
NAIP expression along the cell-cycle. (**a**) NAIP mRNA relative expression in exponentially growing (no treatment) or drug synchronized HeLa cells (400 *μ*M L-mimosine, G1; 2 mM thymidine, S; 0, 4 *μ*g ml^−1^ nocodazole, G2/M). (**b**) NAIP protein levels and representative western blot of non-treated, G1, S and G2/M synchronized HeLa cells. *Significant difference (P < 0.02). Data are the mean ± s.d. of three independent determinations.

Given the unanticipated localization of NAIP to the cytokinetic machinery, we elected to determine whether NAIP is differentially expressed along the cell cycle. NAIP mRNA or protein expression was measured in HeLa cells chemically arrested in G1 with L-mimosine (75%), in S with thymidine (65%) and in G2/M with nocodazole (60%) as determined by DNA-content analysis (data not shown). NAIP mRNA in G2/M was 2.7 fold that of NAIP mRNA in G1 (Fig. 6a), this increase was reflected at the protein level with NAIP two fold higher in G2/M compared to G1 (Fig. 6b).

### The impact of infra and supraphysiologic NAIP on cell phenotype

**Figure 7:**
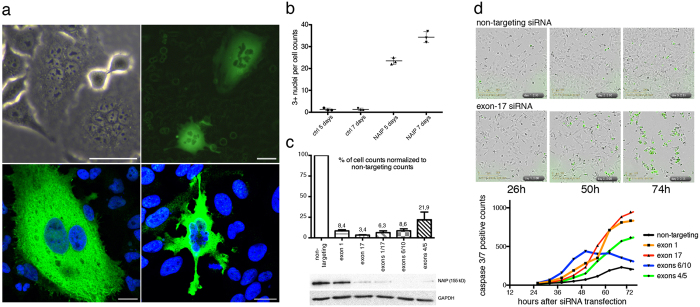
NAIP overexpression and loss of function. (**a**) Upper panel: Phase-contrast and fluorescence live microscopy images of HeLa cells 7 days after transduction with ‘*NAIP* + *neo*’ or ‘*NAIP* + *GFP*’ lentiviral particles respectively. Non transduced cells surrounding the multinuclear fluorescent ‘*NAIP* + *GFP*’ transduced cells show a faint green glow profile. Bar, 50 *μ*m. Lower panel: NAIP immunostaining with abcam ab25968 of HeLa cells (Alexa Fluor-488) 7 days after transduction with ‘*NAIP* + *neo*’ lentiviral particles demonstrating that the multinuclear phenotype is observed in cells overexpressing NAIP. Bar, 20 *μ*m. (**b**) Total visual counts of HeLa cells with three or more nuclei in four randomly selected 10X optical fields from control (non-transduced) and ‘*NAIP* + *neo*’ transduced HeLa cells 5 and 7 days after transduction. Data are the mean ± s.d. of three independent transductions. (**c**) Percentage of cells remaining in culture three days after transfection with various combinations of NAIP siRNA duplexes targeting the indicated NAIP mRNA exons and western blot showing the corresponding efficacy in NAIP silencing. Data are the mean ± s.d. of three independent determinations. (**d**) Upper panels: Phase-contrast and green-fluorescence IncuCyte ZOOM merged images of non-targeting and NAIP exon17-siRNA transfections taken 26, 50 and 74 hours after siRNA transfection. Fluorescent cells show active caspase-3/7, a reflection of the processing of a non-fluorescent caspase-3/7 substrate releasing a green fluorescent dye. Lower graph: Apoptotic cell counts 26 to 74 hours after NAIP siRNA transfection. Each point represents the mean of the caspase-3/7 counts for 4 different optic fields analyzed per well and treatment at a given time point. The graph is representative of one out of four replicas of two independent siRNA transfections.

In an effort to elucidate a role for NAIP in cellular division in light of the protein’s cytokinetic localization, the impact of supraphysiologic NAIP in HeLa cells was next studied. Cells were transduced with a bicistronic lentiviral vector system expressing the open reading frame of NAIP and either the green fluorescent protein (GFP) or the neomycin-resistance (neo) gene. Multinuclear cells could be observed three days after transduction. The multinuclear phenotype, readily detected by phase-contrast and fluorescent live cell microscopy (upper panel in Fig. 7a), was observed in those cells expressing the highest levels of NAIP as determined by immunostaining (lower panel in Fig. 7a). The proportion of HeLa cells with three or more nuclei increased over time with approximately a 30 fold increase observed after a week of NAIP exposure (Fig. 7b). In contrast, NAIP gene silencing by small-interference RNA technology (siRNA) resulted in a lethal apoptotic phenotype (Fig. 7c) for HeLa cells. Apoptosis in dying siRNA transfected cells was demonstrated by monitoring caspase-3 and caspase-7 activity over 48 hours during the second and third day following siRNA transfection (Fig. 7d). On day three after transfection, virtually no cells were left in the plate wells transfected with NAIP siRNA duplexes. NAIP expression silencing was performed using 8 different siRNA duplexes combined in 4 duplexes-pairs altogether targeting at a total of 6 different NAIP mRNA exons; similar cell death were seen with all combinations consistent with this being a true biologic (and not off target) effect.

The main NAIP antibody used in this study (abcam ab25968) has been validated in a number of ways; western blots of HeLa extracts show the anticipated 155-kDa band, and a more intense band of the same size in blots of protein extracts of HeLa’s transiently transfected with a NAIP plasmid vector (data not shown). Similarly, HeLa cells transduced with NAIP-lentiviral particles show strong immunostaining in cells overexpressing NAIP (lower panel in Fig. 7a). Stronger evidence that NAIP is being detected in our analysis comes from the fact that another commercial human-NAIP antibody (Fig. 1d) and a commercial mouse-NAIP antibody (Supplementary Fig. S1) recognize the same structures in HeLa and mouse mASCs cells respectively. Furthermore, a total of three additional custom monoclonal antibodies directed at distinct NAIP epitopes, hybridoma cultured supernatants which regularly do not show NAIP in cytokinesis, demonstrate NAIP in HeLa mitotic cells following transduction with NAIP lentiviral particles. For these reasons we believe our unanticipated mapping of NAIP to the cytokinetic machinery reflects the biologic reality.

## Discussion

We show here by immune localization, the presence of NAIP within subcellular structures associated with cell division. This is the first recorded observation of such a presence; a previous proteomic analysis of human cells did not detect NAIP as an intercellular bridge component^44^; however, the same study also failed to identify well established cytokinesis regulators such as PRC1, the chromosomal passenger components INCENP, Survivin and Borealin, the Centralspindlin MgcRacGAP or BRUCE. Similarly, proteomic analyses of the mitotic spindle in HeLa cells^45^ and, more recently, in Chinese Hamster Ovary cells^46^, although also failing to detect NAIP, did not identify BRUCE, the augmin complex^47^ and Ect2^2^ or KIF4A, BRUCE, Aurora B and the augmin complex respectively, suggesting that as powerful as these approaches are, they are not completely comprehensive.

NAIP is, after Survivin^48^ and the BIR repeat-containing ubiquitin-conjugating enzyme (BRUCE)^49^, the third member of the IAP family to be implicated in cytokinesis. Survivin is one of the components of the CPC and implicated in the regulation of mitotic spindle assembly as well as being involved in the inhibition of apoptosis in the G2/M phase of the cell cycle^50^. BRUCE is involved in abscission, proposed as a platform mediating membrane delivery to the intercellular bridge as well as coordinating multiple steps in abscission through its ubiquitin conjugating activity. Although BRUCE and Survivin provide a precedent for IAP involvement in cytokinesis and NAIP may operate in a functionally similar manner, the sole motif that they share with NAIP, the BIR domain, is phylogenetically quite distantly related^51^. The other defining motif found in NAIP, the Leucine-rich repeat (LRR), comprises the C-terminal half of the protein. A key feature of the pattern recognition receptor NLR protein family^6^,^7^ is the binding to Pathogen-associated molecular patterns (or PAMPs) through their LRR domains, the NAIP-LRR domain binds bacterial flagellin and the needle and rod proteins of the bacterial type-III secretion system PAMPs^11^,^12^,^13^,^14^, these interactions make NAIP an essential component of the NLRC4 inflammasome^15^. LRRs are present in a wide variety of proteins mediating diverse protein-protein interactions and, similarly, diverse functions^52^. Interestingly, in fission yeast^53^ and in the slime mold *Dictyostelium discoidea*^54^, LRR containing proteins have been associated with cytokinesis. Similarly, plant LRR proteins have been linked to cytokinesis as well^55^,^56^,^57^,^58^.

In addition to the novel localization of NAIP to the cytokinetic process, our results bring an increased molecular resolution to the process itself. For instance, at the beginning of cytokinesis, microtubule stabilizing factors, CPC, Centralspindlin and NAIP colocalize in the middle of the central spindle. Later, cleavage furrow ingression and compaction of the central spindle into a ring, shows NAIP in the centermost section of the ring (Fig. 5a-late anaphase arrows) along with Centralspindlin (Fig. 4-anaphase), while PRC1, KIF4A and CPC have segregated to the sides of the medial ring section. Our study highlights this centermost section, precisely defined at the end of the anaphase, as a distinct central spindle area in which NAIP and Centralspindlin are present with PRC1, KIF4A and CPC circumscribing it. The fact that the central spindle intervenes in the definition of the division plane and that Centralspindlin, proposed to link the central spindle to the plasma membrane during cytokinesis^35^, is observed in this region might help setting directions for the study of this medial central spindle zone’s functional significance. Our results are in agreement with the three previously described intercellular bridge regions^40^: the stem body, the bulge and the flanking zone. Interestingly, NAIP and Centralspindlin colocalize in the bulge (outer region of the stem body), in which MgcRacGAP occupies the margins closer to the plasma membrane (Fig. 4, Fig. 5b-NAIP + MgcRacGAP). This is consistent with a role for Centralspindlin in the tethering of the central spindle to the plasma membrane^35^. Finally, our observation of PRC1 immunostained projections extending from the intercellular bridge arms into the nascent daughter cells (Fig. 2a-intercellular bridge, Fig. 5b) has not, to our knowledge, been previously documented; the functional significance of these extensions will clearly require further analysis.

The acquisition of a multinuclear phenotype in HeLa cells overexpressing NAIP is consistent with a functional role for NAIP in cytokinesis; the overexpression of the protein may in some fashion subvert the definition of the division plane. In view of the specific positioning of NAIP to the middle of the central spindle, it may be that supraphysiological-NAIP results in defective formation of the division plane; persistent cell cycling associated with a failure of cytokinesis resulting in large multinucleated cells (Fig. 7a). In contrast, the lethal phenotype observed after NAIP siRNA gene silencing (Fig. 7b) might be due to a pleiotropic loss of function effect; if lethality was uniquely the consequence of dysregulated mitosis, we would anticipate that cell cycle analysis conducted after NAIP siRNA transfection would show a G2/M phase increase reflecting cell death at the end of the cell cycle. Propidium iodide flow cytometry analysis conducted 48 and 72 hours after transfection with all of the siRNA duplexes used in NAIP silencing failed to show this (Supplementary Fig. S5). Previous studies have shown the antiapoptotic effect of NAIP overexpression in cultured cells^5^; it may be, conversely, that the abrupt loss of NAIP confers some degree of apoptotic susceptibility in interphase cells. The involvement of NAIP with cell cycle progression will be investigated in future studies.

Although the images shown are representative of multiple immunostainings performed with the various antibody combinations at different mitotic stages, occasionally, a weaker NAIP immunofluorescence was detected in the flanking areas of the intercellular bridge. Given that this was not consistently observed throughout our study, it was elected to leave this finding out of the general description. Interestingly within atypical mitotic cells, both aberrant (e.g. in the center of an anomalous ring metaphase, Supplementary Fig. S4a) and typical (e.g. on central spindles and intercellular bridges in a polyploid cell undergoing mitosis, Supplementary Fig. S4b) NAIP immunofluorescence was observed.

Proteins required for the M phase machinery and proper M phase progression are typically expressed in the G2 phase of the cell cycle. The 2.7 fold increase in NAIP gene expression observed in G2 versus G1 is consistent with a role for NAIP during the M phase. It may be that NAIP is involved in broader functions and it also has a role in mitosis; the nuclear division process that together with cytokinesis define the M-Phase.

In conclusion, we document a previously unknown localization of NAIP along the entire cytokinetic process whose dynamics exhibits a distinct behaviour (Fig. 1c); the molecular dissection of this novel profile may lead to a better understanding of the final steps of cell division. Future studies might include the investigation of NAIP post-translational modifications and protein variants expression and their relationship with cytokinesis as well as establishing which NAIP protein motifs are required for these putative roles along with the interaction between NAIP with the well established cytokinesis regulators.

## Methods

### Cell culture and synchronization of HeLa cells

HeLa cells (CCL2; American Type Culture Collections, Manassas, Va) and mouse adipose-derived mesenchymal stem cells, mASCs (obtained in the Andalusian Stem Cell Bank (BACM)/University of Granada^59^), were maintained in standard conditions (37 °C in a 5% CO2 humidified atmosphere) in Dulbecco’s modified Eagle’s medium (HyClone DMEM Ca#SH30022.01) supplemented with 10% fetal calf serum (FCS) and 1% antibiotics (100 U/ml penicillin–“streptomycin). For synchronization, HeLa cells were treated for 16 h at 37 °C with 400 *μ*M L-mimosine (Sigma-Aldrich) to arrest cells in G1, 2 *μ*M thymidine (Sigma-Aldrich) to arrest cells in S, or 0, 4 *μ*g ml^−1^ nocodazole (Sigma-Aldrich) to arrest cells in G2/M.

### Immunostaining and microscopy

HeLa cells were grown in 8-well glass chamber slides for confocal microscopy (Nunc Lab-Tek Ca#177402) and 2-well chambered coverglass for STED super resolution microscopy (Nunc Lab-Tek II Ca#155379); the cells were fixed for 15 minutes in ice-cold 4% paraformaldehyde in PBS, briefly rinsed in PBS and permeabilized with 0.2% Triton X-100/PBS for 10 minutes. Permeabilization solution was next aspirated and the slides were then incubated during 2 hours at room temperature with primary antibodies appropriately mixed and diluted in PBS (references and dilutions listed below), slides were then rinsed 3 times for 5 minutes with PBS and incubated for 45 minutes at room temperature with the appropriate secondary antibodies, goat anti-mouse Alexa Fluor 488 and goat anti-rabbit Alexa Fluor 568 or rabbit anti-goat Alexa Fluor 568 (Invitrogen, A-11001, A-11036 and A-11079) diluted at 1:1000 in PBS. The slides were then rinsed 3 times for 5 minutes with PBS and mounted with ProLong Gold (Invitrogen). During the second wash, the slides were counterstained for 5 minutes with Hoechst 33342 (Invitrogen) diluted at 10 *μ*g/ml in PBS. For STED microscopy, the secondary antibodies were incubated for 45 minutes at room temperature, Chromeo 505 anti-rabbit (Active Motif) diluted at 1:2000 in PBS and Biotin anti-mouse (Sigma-Aldrich) diluted at 1:300 in PBS, then rinsed 3 times for 5 minutes with PBS and incubated for 30 minutes at room temperature with Streptavidin (BD Horizon) diluted at 1:200 in PBS, rinsed 3 times for 5 minutes with PBS and mounted with ProLong Gold (Invitrogen). Confocal microscopy was performed with an Olympus FluoView FV1000 microscope and dual colour STED microscopy was performed with a Leica TCS SP5-STED CW microscope. STED Images were processed using Leica STED deconvolution software (LAS AF v2.6.3.8173). First a point spread function (PSF) was generated using a Lorentz transformation of 70 nm. Then the image was deconvolved using the generated PSF and signal energy set to regularization parameter 0.05. Images were further processed using the baseline mean function, and background was reduced by 1500 (in the 16-bit image).

Primary commercial NAIP antibodies and dilutions used in this study: abcam ab25968; epitope mapping to 13 C-terminal residues of human NAIP (1.4:1000 and 1:250 in HeLa and mASCs immunostainings respectively). abcam ab98020; epitope mapping to the first 1–100 residues of human NAIP (1:200). Santa Cruz Biotech, Inc. sc-11064; epitope mapping near the C-terminus of NAIP5 of mouse origin (1:25).

Custom NAIP monoclonal hybridoma supernatants developed by Medical & Biological Laboratories Co., Ltd. used in this study: **(a)** epitope mapping to residues 961-970 (ERNLAEKEDN) of human NAIP (NP_004527.2), **(b)** epitope mapping to residues 1304-1313 (KITEEGYRNF) of human NAIP (NP_004527.2) and **(c)** epitope mapping to residues 324-333 (KWQEGDDPLD) of human NAIP (NP_004527.2).

Other primary commercial antibodies and dilutions used in this study: abcam Aurora B #ab3609 (1:150) and *α*-Tubulin #ab7291 (1:500). Santa Cruz Biotechnoly, Inc. INCENP #sc-376514 (1:150), Survivin #sc-17779 (1:100), Rac GAP1 #sc-271110 (1:50), PRC1 #sc-376983 (1:50) and KIF4 #sc-365144 (1:50).

### Quantitative PCR

Total RNA was extracted from cells with Trizol reagent (Invitrogen) as recommended by the supplier. cDNA was obtained using the *Promega Reverse Transcription System* kit according to manufacturer’s instructions. The synthesized cDNA was used for real-time PCR employing the *FastStart SYBR Green Master* (Roche) and analyzed with the Eppendorf *Mastercycler RealPlex*^2^ using the *Realplex* software. Real-time PCRs were done using the following primers: NAIP, forward 5′GAATTTATCGAGTGGCCAAAC3′ and reverse 5′TCAAAGACTTGACTGTTGTGG3′; Control primers: human actin, forward 5′TGACGGGGTCACCCACACTGTGCCCATCTA3′ and reverse 5′CTAGAAGCATTTGCGGTGGACGATGGAGGG3′; human HPRT1 forward 5′TGACACTGGCAAAACAATGCA3′ and reverse 5′GGTCCTTTTCACCAGCAAGCT3′; human GAPDH forward 5′TGCACCACCAACTGCTTAGC3′ and reverse 5′GGCATGGACTGTGGTCATGAG3′.

### Western blot analysis

Cells were washed 2 times with ice-cold PBS and lysed in RIPA buffer containing 10 mg/ml of aprotinin, PMSF, and leupeptin (Sigma-Aldrich), 5 mM *β*-glycerophosphate, 50 mM NaF, and 0.2 *μ*M sodium orthovanadate for 30 minutes at 4 °C, followed by centrifugation at 13.000 g for 15 minutes; supernatants were then collected and kept frozen at −20 °C. Total protein concentrations were determined by Bradford protein assay using a Bio-Rad protein assay kit. Standardized amounts of protein samples (25 *μ*g to 50 *μ*g) were separated by 10% SDS-PAGE. Proteins were subsequently transferred onto nitrocellulose membrane and incubated in blocking solution (PBS, 5% nonfat milk, 0.2% Tween-20) for 1 hour at room temperature followed by overnight incubation with the NAIP-J2^36^ rabbit polyclonal antibody at 4 °C diluted at 1:1500. Membranes were washed with PBS-T (PBS, and 0.2% Tween-20) 3 times followed by incubation with secondary antibody (anti-rabbit or mouse; Cell Signaling) for 1 hour at room temperature at the dilution suggested by the manufacturer. Loading control antibody complexes were visualized by autoradiography using the ECL Plus and ECL Western Blotting detection systems (GE Healthcare). Quantification was performed by scanning the autoradiographs, and signal intensities were determined by densitometry analysis using the ImageJ program (National Institutes of Health, USA).

### Lentiviral transduction

The lentiviral vector system used was as previously described^60^. Plasmids construction. NAIP transfer vectors were constructed by standard cloning techniques; BamHI-AsiSI restriction enzymes were used to replace the p27 cDNA in the Cp27WP plasmid (a SIN-LV plasmid expressing p27 through the CMVTetO promoter, F. Martin’s lab, unpublished) with the NAIP insert obtained by PCR from OriGene SC303496 cDNA clone, the resulting plasmid was called cNAIP-WP. To generate cNAIP + Neo-WP and cNAIP + GFP-WP, a pGEMT/XhoI-neo insert (expressing the Neo resistance gene through the PGK promoter) and a blunted PstI 1.2Kb fragment (expressing eGFP through the Spleen Fcus Forming Virus (SFFV) LTR promoter) from the CTetOVSEWP plasmid (F. Martin’s lab, unpublished) were ligated to the opened XhoI and XhoI-blunted cNAIP-WP respectively (vector maps in Supplementary information). The resulting vectors were sequenced and confirmed to be error free and in-frame as designed. Lentiviral particles production. Briefly, 293 T cells cultured in 10 cm dishes were transfected with 8 *μ*g of transfer, packaging and envelope vectors (plasmid proportion 4:3:1) diluted in 1.5 ml of Opti-MEM (GIBCO) and mixed with 20 *μ*l of X-tremeGene HP (Roche). Culture media with the DNA mix was replaced with fresh media 24 hours after transfection, the supernatant with lentiviral particles was harvested and filtered (0.45 *μ*m pore size) 48 hours after transfection started. Transduction. One ml of readily collected lentiviral particles was added to HeLa cells grown in 6-well plates and replaced with fresh media 24 hours after transduction was initiated.

### siRNA transfection and survival analysis

NAIP siRNA silencing was performed in 24-well plates. Briefly, NAIP siRNA-duplexes (siRNA sequences listed in Supplementary information) were transfected at a final 10 nM concentration: 50 *μ*l of 80 nM siRNA and 50 *μ*l of 2X/Opti-MEM (GIBCO) with 0, 25 *μ*l of Lipofectamine RNAiMAX (Invitrogen) were plated per well and left to mix during 20 minutes, 300 *μ*l of media with cells were then added per well. 72 hours after siRNA transfection, the cells the were fixed, stained with Hoechst 33342 (Invitrogen) and 4 fields/well were imaged with a 5x objective on a Cellomics Arrayscan Vti automated 2D fluorescence microscope. Cell nuclei counts were measured from the images using Cellomics Scan Software.

Apoptosis in siRNA transfected cells was monitored with the IncuCyte ZOOM live-cell imaging system (Essen Bioscience). An activated-caspase substrate was added 24 hours after siRNA transfection (1:5000, IncuCyte Caspase-3/7 Reagent, Essen BioScience), 4 phase-contrast and green fluorescent images of the same optic field were automatically taken per hour in each well over two days. Analysis of apoptotic cells was performed using the IncuCyte image analysis tools software (Essen BioScience).

## Additional Information

**How to cite this article**: Abadía-Molina, F. *et al*. Neuronal apoptosis inhibitory protein (NAIP) localizes to the cytokinetic machinery during cell division. *Sci. Rep.* **7**, 39981; doi: 10.1038/srep39981 (2017).

**Publisher's note:** Springer Nature remains neutral with regard to jurisdictional claims in published maps and institutional affiliations.

## Supplementary Material

Supplementary Information
