# Supplementary material for: Neuronal apoptosis inhibitory protein (NAIP) localizes to the cytokinetic machinery during cell division
Source: Sci Rep. 2017 Jan 6;7:39981. doi: 10.1038/srep39981 (PMC5216396; doi:10.1038/srep39981)
Supplement: Supplementary Information [file srep39981-s1.pdf]

Neuronal apoptosis inhibitory protein (NAIP) localizes to the cytokinetic machinery during cell division

Francisco Abadía-Molina[1,2], Virginia Morón-Calvente[1,2], Stephen D. Baird[3], Fahad Shamim[3], Francisco Martín[4], Alex MacKenzie[3,5].

1. Department of Cell Biology, University of Granada, Granada 18071, Spain
2. Biomedical Research Centre, University of Granada, Granada 18100, Spain
3. Children's Hospital of Eastern Ontario Research Institute, University of Ottawa, Ottawa ON K1H 8L1, Canada
4. Department of Human DNA Variability, GENYO, Centre for Genomics and Oncological Research, Pfizer, University of Granada, Andalusian Regional Government, Granada, 18016 Spain
5. Department of Pediatrics, University of Ottawa, Ottawa ON K1H 8L1, Canada

Supplementary information

Figure S1. NAIP in mouse Adipose-derived mesenchymal Stem Cells (mASCs) cytokinesis showed with two NAIP antibodies.

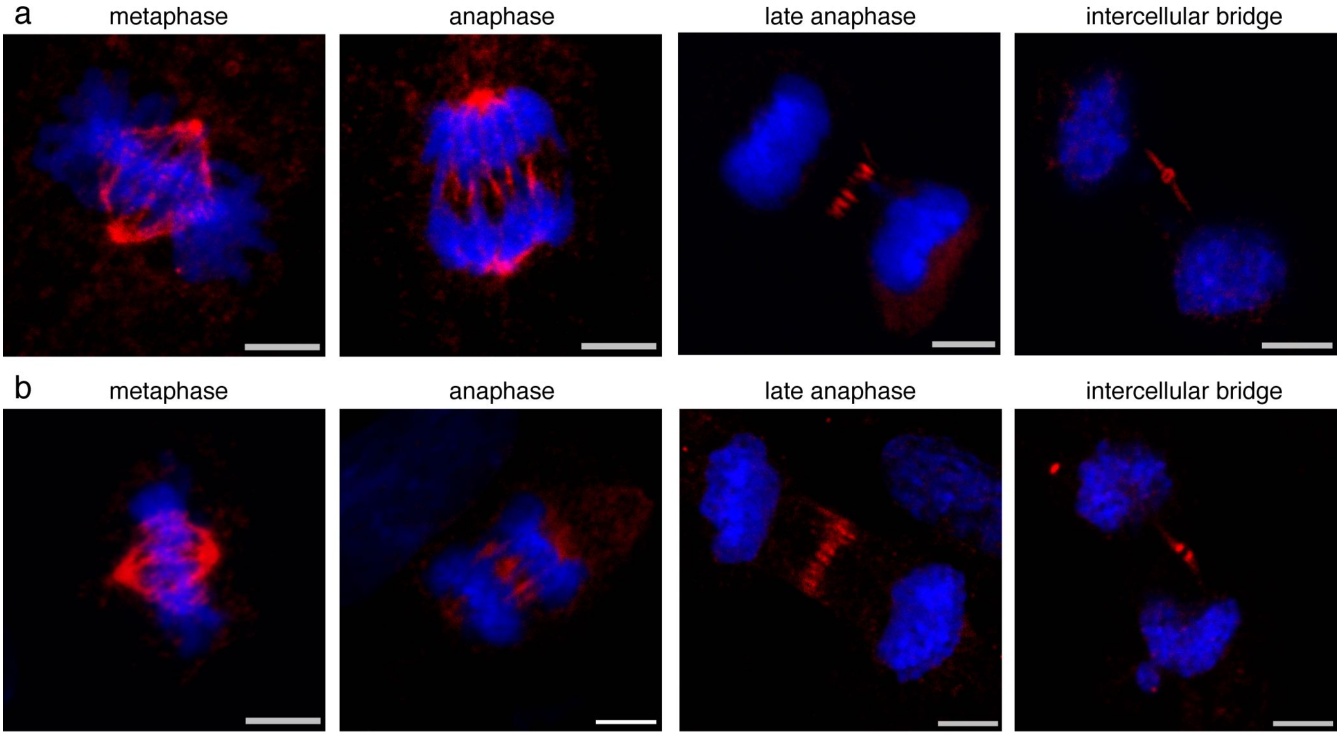

Nuclear Hoechst staining and NAIP immunofluorescence (Alexa Fluor-568) showing NAIP in metaphase, anaphase, late anaphase and in the intercellular bridge of mitotic mASCs cells. **(a)** NAIP antibody from abcam® (ab25968, epitope mapping to 13 C-terminal amino acids of human NAIP and predicted to react with mouse NAIP, 1:250) demonstrating NAIP in mASCs mitotic images. **(b)** NAIP antibody from Santa Cruz Biotechnology, Inc. (sc-11064, epitope mapping near the C-terminus of NAIP5 of mouse origin, 1:25) demonstrating NAIP in mASCs mitotic images. Bar, 5µm.

Figure S2. NAIP in cytokinesis showed with monoclonal antibodies from three different NAIP hybridoma supernatants in HeLa cells transduced with NAIP-lentiviral particles.

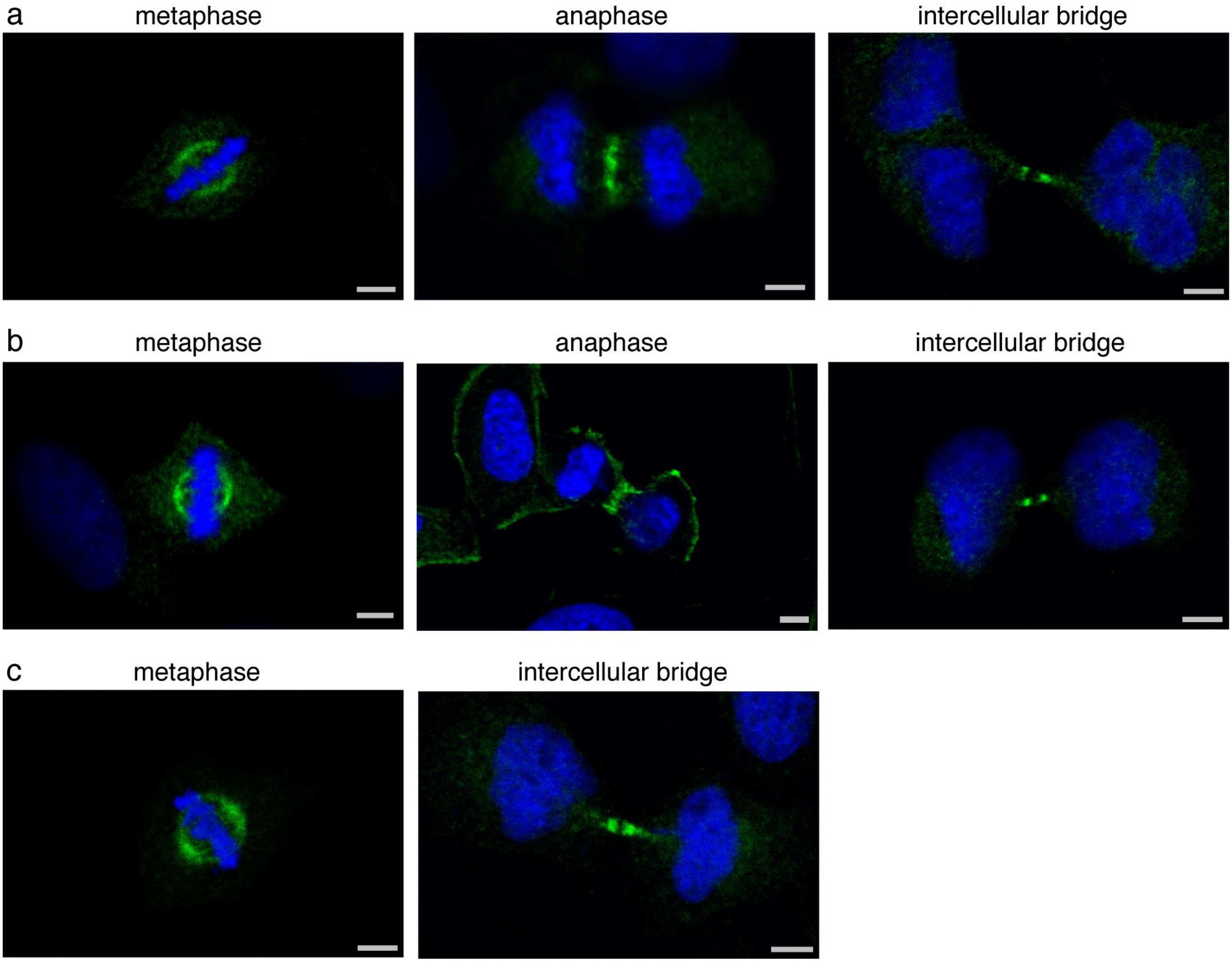

Nuclear Hoechst staining and NAIP immunofluorescence (Alexa Fluor-488) showing NAIP in mitotic HeLa cells. IF was performed 4 days after transduction with *‘NAIP+neo’* lentiviral particles. **(a)** Custom monoclonal hybridoma supernantant mapping to residues 961 to 970 of human NAIP (NP_004527.2) showing NAIP in metaphase, anaphase and in the intercellular bridge. **(b)** Custom monoclonal hybridoma supernantant mapping to residues 1304 to 1313 of human NAIP (NP_004527.2) showing NAIP in metaphase, anaphase and in the intercellular bridge. **(c)** Custom monoclonal hybridoma supernantant mapping to residues 324 to 333 of human NAIP (NP_004527.2) showing NAIP in metaphase and in the intercellular bridge. Bar, 5µm.

Figure S3. Survivin and NAIP immunostaining in HeLa cells.

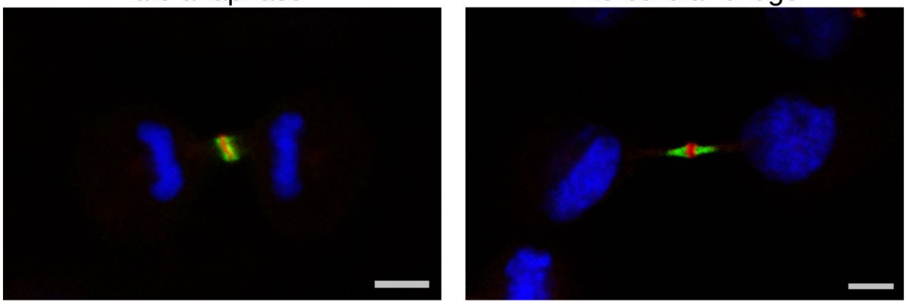

Double immunostaining for survivin (Alexa Fluor-488 green fluorescence) and NAIP ab25968 (Alexa Fluor-568 red fluorescence) showing survivin in late anaphase and in the intercellular bridge equivalently to the other chromosomal passenger complex components analyzed. Bar, 5µm.

Figure S4. Aberrant metaphase and polyploid HeLa mitosis immunostaining.

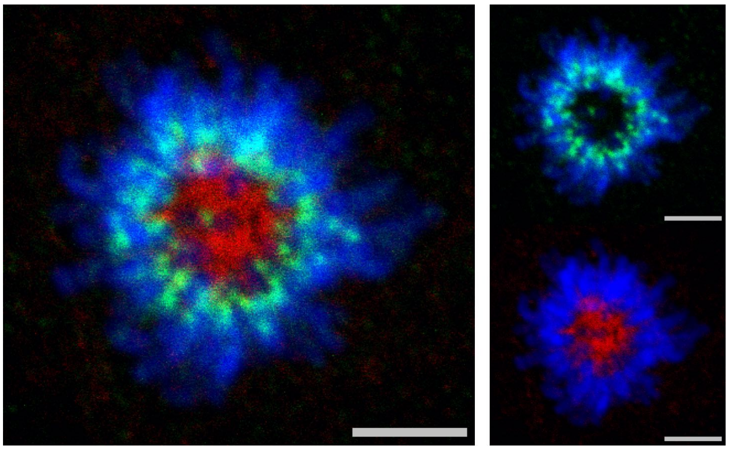

(a) Double immunostaining for INCENP (Alexa Fluor-488 green fluorescence) and NAIP ab25968 (Alexa Fluor-568 red fluorescence) showing INCENP in the chromosome centromeres in an aberrant ring metaphase, interestingly NAIP occupies the center of the metaphasic ring where an irregular mitotic spindle should be present. Bar, 5µm.

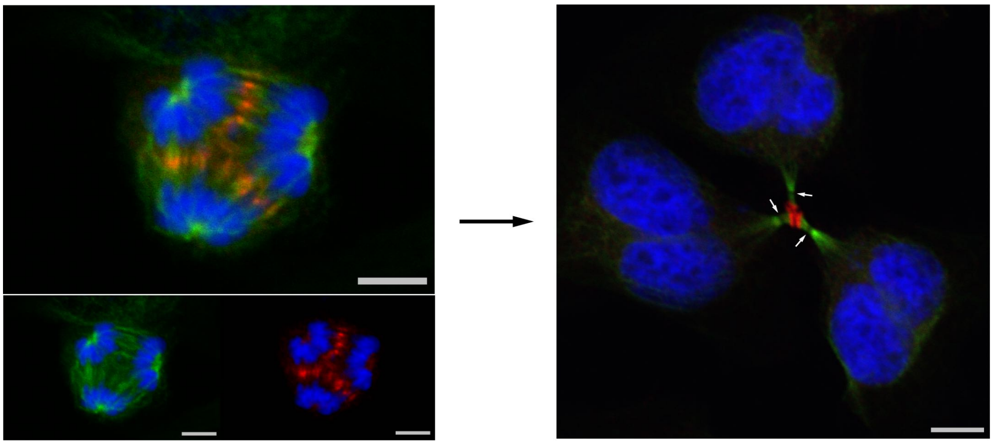

(b) Double immunostaining for α-Tubulin (Alexa Fluor-488 green fluorescence) and NAIP ab25968 (Alexa Fluor-568 red fluorescence) in a polyploid HeLa cell mitosis showing a double cytokinetic apparatus. In anaphase two distinct central spindles are organized, after cell division completion two stem bodies and three abscission points (arrows) in the corresponding intercellular bridges are present. Bar, 5μm.

Figure S5. Cell cycle analysis in NAIP siRNA silenced HeLa cells.

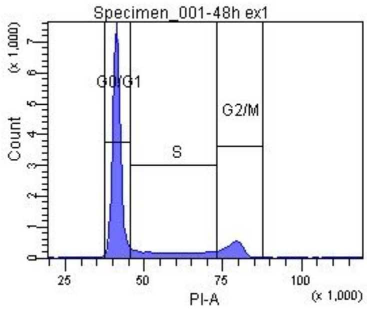

48h exon 1 siRNA

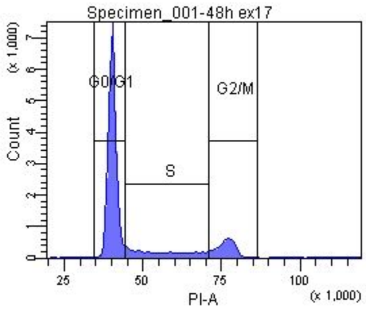

48h exon 17 siRNA

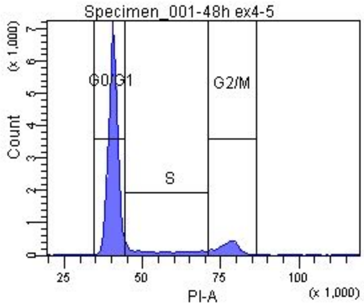

48h exon 4/5 siRNA

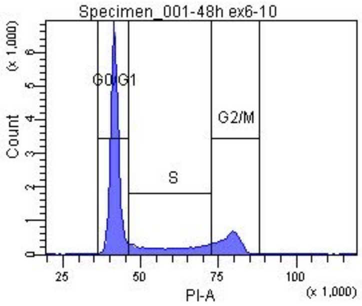

48h exon 6/10 siRNA

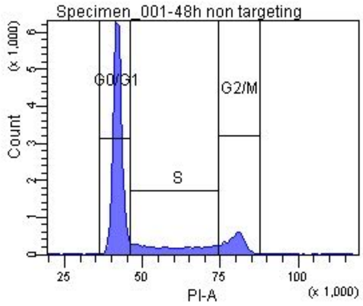

48h non-targeting siRNA

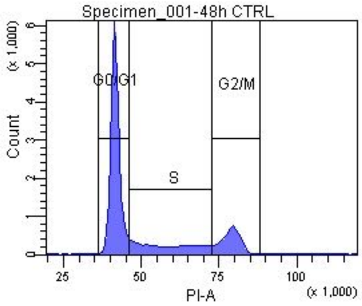

48h non-treated

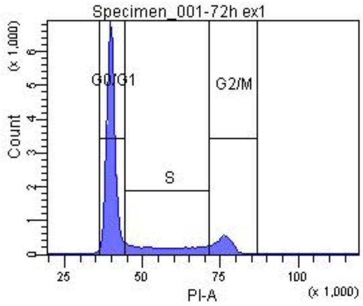

72h exon 1 siRNA

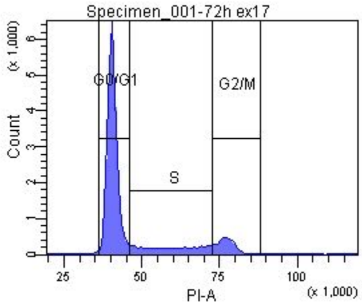

72h exon 17 siRNA

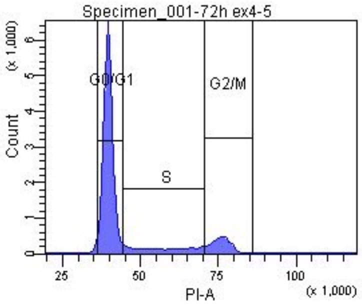

72h exon 4/5 siRNA

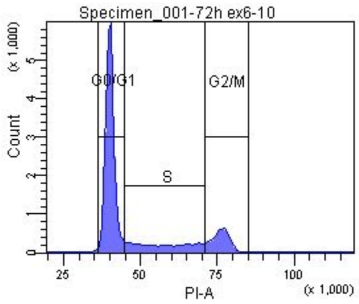

72h exon 6/10 siRNA

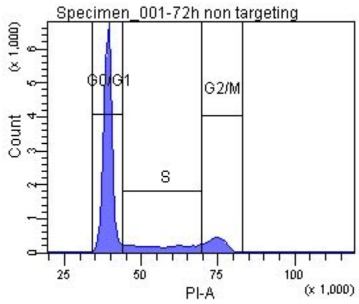

72h non-targeting siRNA

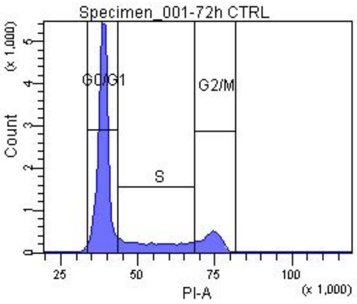

72h non-treated

Propidium iodide cell cycle analysis of HeLa cells transfected with different combinations of NAIP siRNA duplexes targeting the indicated NAIP mRNA exons 48 and 72 hours after transfection. No substantial changes in cell cycle phase percentages were detected between treatments for the remaining adherent cells after transfection. Efficiency of the transfection was monitored using a human cell death phenotype control siRNA (*QIAGEN AllStars Hs Cell Death siRNA*) that showed a marked G2/M cell cycle increase 24 and 48 hours after transfection (66,6% and 61.2% respectively). Graphs are representative of three independent experiments.

Propidium iodide staining: adherent HeLa cells were lifted up and a pellet was obtained after centrifugation at 300 x g for 5 minutes, the cells were then resuspended in 100µl of PBS and

900µl of ice cold 78% ethanol were added and left 5 minutes, a cellular pellet was obtained by centrifugation after fixation, washed once with PBS and resuspended in 250µl of a propidium iodide (40µg/ml, *Sigma-Aldrich*) RNase (100µg/ml, *Invitrogen RNase A*) solution and incubated at 37°C for 30 minutes before taking them to the flow cytometer.

NAIP transfer vector maps:

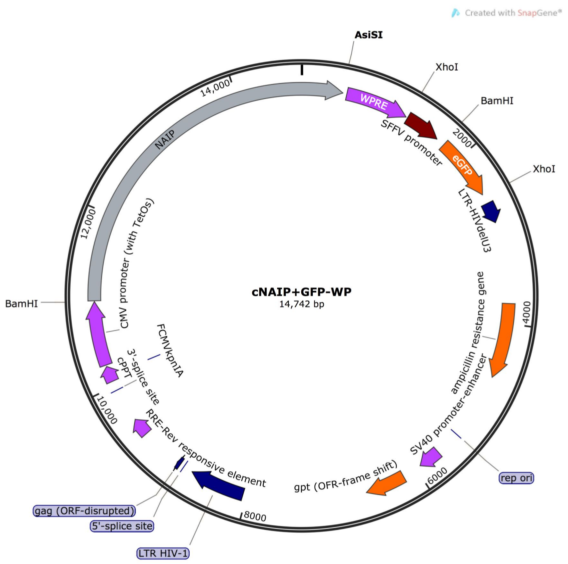

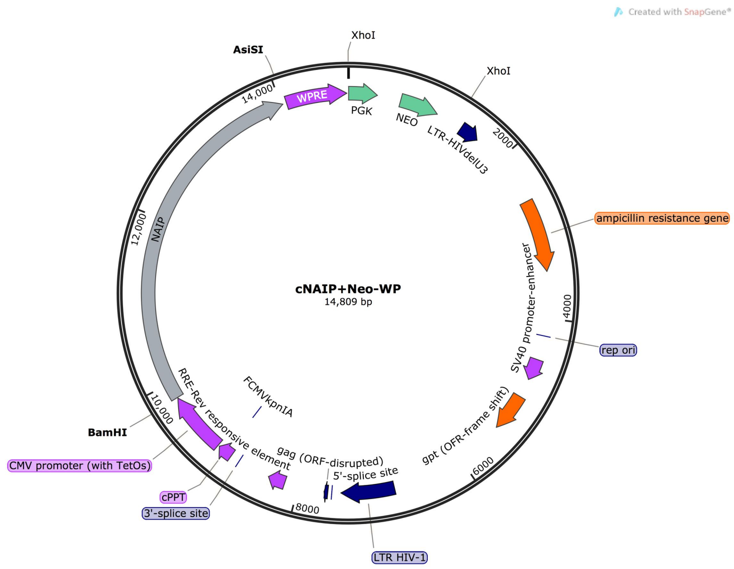

NAIP sense strand siRNA sequences:

Exon 1; 5'-GAUUAGAGGUCUGGGAUUUUU-3' and 5'-CCACAGGUUUGGAGAGCUUUU-3'

Exon 17; 5'-CUGCCAAUAUAAAGAGGAAUU-3' and 5'-AGUAUGAAAUAUAGGGACAUU-3'

Exon 4; 5'-CAAAGGAACUAGAAGAAGAUU-3'

Exon 5; 5'-UGGUGGAUGUUUAGGAAAUUU-3'

Exon 6; 5'-GUGAAUUUCUUCGGAGUAAUU-3'

Exon 10; 5'-GGAAGUGACUCCAGACCUUUU-3'

Non-targeting: *QIAGEN Negative Control siRNA*.

Sequences were chosen using the *Dharmacon siDESING Center* tool.
